# Preoperative single ventricle function determines early outcome after second-stage palliation of single ventricle heart

**DOI:** 10.1186/s12947-017-0114-7

**Published:** 2017-09-11

**Authors:** Jacek Pająk, Michał Buczyński, Piotr Stanek, Grzegorz Zalewski, Marek Wites, Lesław Szydłowski, Bogusław Mazurek, Lidia Tomkiewicz-Pająk

**Affiliations:** 1 Pediatric Heart Surgery and General Pediatric Surgery Department, Medical University of Warsaw, ul. Żwirki i Wigury 63A, 02-091 Warszawa, Poland; 2 Pediatric Heart Surgery Department, The Independent Public Clinical Hospital no. 6 of the Medical University of Silesia, Katowice, Poland; 3 Department of Pediatric Cardiology, Medical University of Silesia, Katowice, Poland; 4 Institute of Cardiology, Jagiellonian University, Medical College and John Paul II Hospital, Krakow, Poland

**Keywords:** Second-stage single-ventricle palliation, Single-ventricle heart, Hemi-Fontan, bidirectional Glenn procedure, Hypoplastic left heart syndrome, Extracellular matrix, CorMatrix

## Abstract

**Background:**

Second-stage palliation with hemi-Fontan or bidirectional Glenn procedures has improved the outcomes of patients treated for single-ventricle heart disease. The aim of this study was to retrospectively analyze risk factors for death after second-stage palliation of single-ventricle heart and to compare therapeutic results achieved with the hemi-Fontan and bidirectional Glenn procedures.

**Material and methods:**

We analyzed 60 patients who had undergone second-stage palliation for single-ventricle heart. Group HF consisted of 23 (38.3%) children who had been operated with the hemi-Fontan method; Group BDG consisted of 37 (61.7%) who had been operated with the bidirectional Glenn method. The analysis focused on 30-day postoperative mortality rates, clinical and echocardiographic data, and early complications.

**Results:**

The patients’ ages at the time of repair was 33 ± 11.2 weeks; weight was 6.7 ± 1.2 kg. The most common anatomic subtype was hypoplastic left heart syndrome, in 36 (60%) patients. The early mortality rate was 13.3%. Significant preoperative atrioventricular valve regurgitation, single-ventricle heart dysfunction, pneumonia/sepsis, and arrhythmias were associated with higher mortality rates after second-stage palliation. Multivariate analysis identified significant preoperative single-ventricle heart dysfunction as an independent predictor of early death after second-stage palliation. No differences were found in the analyzed variables after bidirectional Glenn compared with hemi-Fontan procedures.

**Conclusion:**

Significant preoperative atrioventricular valve regurgitation, arrhythmias and pneumonia/sepsis are closely correlated with mortality in patients with single-ventricle heart after second-stage palliation. Preoperative significant single-ventricle heart dysfunction is an independent mortality predictor in this group of patients. There are no differences in clinical, echocardiographic data, or outcomes in patients treated with the hemi-Fontan compared with bidirectional Glenn procedures.

## Background

Second-stage palliation, using the hemi-Fontan or bidirectional Glenn procedures in the surgical treatment of single-ventricle heart has reduced the complication rate and improved outcomes after the final stage, i.e. the Fontan operation. Anatomically, second-stage palliation for single-ventricle heart represents one-half of systemic venous-to-pulmonary arterial anastomosis, while hemodynamically it leads to normalization of the volume load of the single ventricle [1–3]. Such an intermittent stage promotes better tolerance and gradual transition to the hemodynamic model after the Fontan operation. Second-stage palliation of single-ventricle heart performed with the hemi-Fontan method consists of anastomosing the superior vena cava (SVC) with the pulmonary arteries close to the SVC insertion to the right atrium, while the SVC insertion is separated from the right atrial cavity by means of a transverse patch sutured to the right atrial walls. Such a location of the incision line, anastomosis and patch suturing lines, and future scar formation in this region, pose a risk of damaging the sinus node and/or impulse conduction pathways from the sinus node. These issues may lead to arrhythmias, a severe complication, given the post-Fontan operation circulation physiology [4]. Performing hemi-Fontan as second-stage palliation necessitates performing the Fontan operation, using the “lateral tunnel” technique, which consists of suturing a patch inside the right atrium that directs flow from the vena cava to the pulmonary arteries. Thus, hemi-Fontan does not allow for a selection of the Fontan operation technique to match the anatomy of a defect [5].

In 2011, we decided to switch our second-stage palliation surgical technique from the hemi-Fontan to the bidirectional Glenn. In bidirectional Glenn, the SVC is anastomosed with the pulmonary arteries at a distance from the sinus node region and conduction pathways, an arrangement that would seem to decrease the risk of arrhythmias developing. However, no reports have been published that unambiguously favor either of the methods. Hence, we are our attempting to evaluate hemi-Fontan and bidirectional Glenn based on our clinical outcomes. Both in the hemi-Fontan and bidirectional Glenn technique, achieving a wide anastomosis between the SVC and the pulmonary arteries may require using a biological or artificial patch to enlarge the anastomosed site. Since the beginning of 2013, we have placed an extracellular matrix (ECM) patch, derived from porcine small intestinal submucosa [6, 7], in all children with single-ventricle heart operated for enlargement of pulmonary artery anastomoses.

The aim of the present study was to retrospectively analyze risk factors of mortality after second-stage palliation of single-ventricle heart, and to compare the therapeutic results achieved with hemi-Fontan and bidirectional Glenn procedures.

## Methods

We conducted a retrospective review of all the patients who had undergone second-stage palliation for single-ventricle heart in the Pediatric Heart Surgery Department (University School of Medicine in Katowice, Poland) between 2003 and 2015. The study protocol was approved by the local ethics committee. Depending on the method of surgical treatment, the patients were assigned to one of two groups. Group HF consisted of 23 (38.3%) children, in whom second-stage palliation had been performed using the hemi-Fontan method in the years 2003–2011. Group BDG consisted of 37 (61.7%) children who had been treated in the years 2011–2015, with the bidirectional Glenn procedure. In this group, 15 (40.5%) patients had a direct end-to-side anastomosis made between the SVC and the right pulmonary artery, while the remaining 22 (59.5%) patients had the SVC and right pulmonary artery anastomosis extended by use of an ECM patch (CorMatrix®; Cardiovascular, Inc., Roswell GA, USA).

Patient demographics, clinical characteristics, imaging, operative reports, hospital records, and clinical reports were collected, and a retrospective analysis of the data was performed.

In all the patients, anatomical details were determined, and the children were deemed qualified for surgical treatment based on echocardiographic findings. Echocardiograms were interpreted by two readers, who assessed the single-ventricle morphology and function and atrioventricular valve function. The single-ventricle function was assessed semi-quantitatively according to the following scale: 1, good; 2, fair; 3, decreased, and 4, poor [8, 9]. Significant impairment of the single-ventricle heart function was defined as a score of more than fair. Semi-quantitative assessment was also used in evaluating valvular competence, the scale being 0, none; 1, mild; 2, moderate; 3, severe [8–10]. Significant regurgitation was defined as a score more than mild. The examinations were performed before operation and on day 2 postoperatively.

The analyses performed in the two groups included postoperative 30-day mortality rates and these variables: anatomy of the defect; age; body mass; aortic clamp time; oxygen arterial blood saturation (Sat O_2_) on day 1, 3 and 5 postoperatively; pneumonia or sepsis; atrioventricular valve regurgitation (AVVR); single-ventricle function; arrhythmias; intubation time; duration of hospitalization; and relations between these variables and their effect on the outcomes.

The diagnosis of a clinically significant arrhythmia in the perioperative period was based on the Cardiosurgical Postoperative Intensive Care Unit monitoring system and a review of all available electrocardiograms. “Arrhythmia” was defined as a rhythm requiring treatment with an antiarrhythmic medication or pacing, or led to cardiopulmonary resuscitation.

### Statistical methods

To assess differences between the groups in qualitative data, we employed the chi-square test or the Fisher’s exact test, whereas quantitative data were evaluated with the t-Student test or Mann-Whitney test. Correlation of quantitative data was analyzed by the Spearman’s correlation coefficient (r_s_); for qualitative data, we employed the chi-square or the Fisher’s exact test. The stepwise logistic regression was used to determine factors affecting postoperative mortality rates.

### Surgical technique

In all patients in both groups, the procedure was performed through a median sternotomy. The ascending aorta was cannulated. In 10 (27.0%) patients in Group BDG, two venous cannulas were inserted, one into the right atrium and another high in the SVC. In these patients, a direct SVC-right pulmonary artery anastomosis was performed with the patient in moderate hypothermia (approximately 32^o^ C). In the remaining children from both groups, a single venous cannula was inserted into the right atrium, and the procedure was performed in deep hypothermia (approximately 18^o^ C) with low flow (cardiac output approx. 50 ml/kg) or with cardiac arrest; crystalline cardioplegia was administered.

In Group HF, all children had a hemi-Fontan anastomosis constructed between the SVC and the pulmonary arteries, and an oval polytetrafluorethylene patch was sutured below the SVC outlet to the right atrium, which separated the SVC outlet from the remaining RA. The anterior part of the SVC-PAs anastomosis was enlarged by use of a homogenous pulmonary artery patch.

**Fig. 1:**
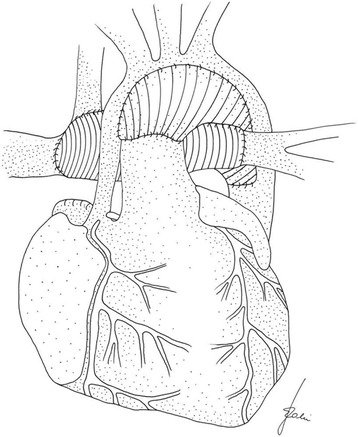
Pulmonary arteries reconstruction with ECM (CorMatrix®) tube in bidirectional Glenn anastomosis. The ECM patch is used for aortic arch reconstruction in the stage I Norwood operation

In Group BDG, 5 (13.5%) children had a direct bidirectional Glenn anastomosis performed in deep hypothermia with low flow. The remaining 22 patients (59.5%) had a modified Norwood I procedure (aortic arch ECM patch reconstruction and bilateral pulmonary artery banding), in which the pulmonary arteries were dissected from the main pulmonary artery trunk, the stumps were proximally closed with sutures, and the junction between the pulmonary arteries and SVC was reconstructed with an ECM (CorMatrix®) tube larger in diameter than the pulmonary artery diameter (Fig. 1). No patient had a surgical correction because of insufficient tricuspid valve.

Postoperatively, all the patients were hospitalized in the Cardiosurgical Postoperative Intensive Care Unit. All patients received heparin in the early postoperative period, then antiplatelet medication until the Fontan operation was performed.

## Results

Patients characteristics are reported in Tables 1 and 2. All children with HLHS operated on in the years 2003–2013 received the Norwood I procedure as modified by Sano et al. [11] as the first-stage operation (part of Group HF). Since 2013, we have routinely used our modification of the Norwood procedure in the first-stage of HLHS treatment; the modification consists of reconstructing the aortic arch with an ECM patch and in bilateral pulmonary arteries banding (part of Group BDG). Patients with diagnoses other than HLHS had made earlier main pulmonary artery banding or systemic – pulmonary anastomosis as a first stage operation or they required no operation.

**Table 1:** Comparison of patients with single-ventricle heart operated on with the hemi-Fontan (Group HF) or bidirectional Glenn (Group BDG) procedure

		Group HF (*n* = 23) m (min-max, %)	Group BDG (*n* = 37) m (min/max, %)	*p* value
Age 33 ± 11.2 (weeks)		28 (10–42)	36 (19–72)	0.03
Body mass 6.7 ± 1.2 (kg)		6 (4–9)	7 (4–10)	0.19
Mechanical ventilation (days)		4 (2–25)	4 (1–20)	0.46
Type of single-ventricle	HLHS	13 (56.5%)	23 (62,2%)	0,66
	TA	2 (8,7%)	4 (10,8%)	0,99
	Unbalanced A-V canal	2 (8,7%)	3 (8,1%)	0,99
	DILV	1 (4,3%)	3 (8,1%)	0,99
	Other	5 (21,7%)	4 (10,8%)	0,28
Aortic clamping time (min)		39 (27–58)	39 (21–61)	0.62
Mechanical ventilation (days)		4 (2–25)	4 (1–20)	0.46
SatO_2_ (%) - 1 postoperative day		75 (40–86)	77 (61–92)	0.96
SatO_2_ (%) - 3 postoperative day		78 (63–85)	80 (65–92)	0.64
SatO_2_ (%) - 5 postoperative day		78 (70–87)	81 (60–91)	0.642
Length of stay (days)		11 (5–19)	10 (7–18)	0.42

*M* median, *SatO2* oxygen arterial blood saturation, *HLHS* hypoplastic left heart syndrome, *TA* tricuspid atresia, *DILV* double inlet left ventricle

**Table 2:** Echocardiographic data, complication and outcome in patients with SV operated on employing the hemi-Fontan and bidirectional Glenn procedures

	Group HF (*n* = 23) n (%)	Group BDG (*n* = 37) n (%)	Total (*n* = 60) n (%)	*p* value
Postoperative Arrhythmias	5 (22)	4 (11)	9 (15.0)	0.28
Postoperative Sepsis	2 (9)	4 (11)	6 (10)	0.99
Preoperative AVVR (0 + 1)	18 (78)	30 (81)	48 (80)	0.89
Preoperative AVVR (2 + 3)	5 (22)	7 (19)	12 (20)	
Postoperative AVVR (0 + 1)	20 (87)	33 (89)	53 (88)	0.79
Postoperative AVVR (2 + 3)	3 (13)	4 (11)	7 (12)	
Preoperative SV function (1 + 2)	19 (83)	31 (84)	50 (83)	0.99
Preoperative SV function (3 + 4)	4 (17)	6 (16)	10 (17)	
Postoperative SV function (1 + 2)	20 (87)	33 (89)	53 (88)	0.99
Postoperative SV function (3 + 4)	3 (13)	4 (11)	7 (12)	
Death	4 (17,4)	4 (10,8)	8 (13,3)	0,464

*AVVR* atrioventricular valve regurgitation, *SV* single ventricle

Table 1 illustrates that Group BDG patients were significantly older than Group HF patients at the time of operation [36 weeks (range 19–72) vs. 28 weeks (range 10–42); *p* = 0.03]. Otherwise, no significant differences were noted in body mass, intubation time, aortic clamp time, arterial blood saturation, duration of hospitalization, arrhythmias, pneumonia/sepsis, mortality rate, AVVR, or systemic ventricular function.

### Risk factors and mortality (Table 2)

#### Postoperative arrhythmias

Postoperative arrhythmias were recorded in 9 of the 60 (15%) children, 5 (22%) in Group HF; 3 (13.0%) of these children had slow sinus rhythm, and 2 (9%) had sinus bradycardia. Among the 4 (11%) in Group BDG, 1 (3%) had slow sinus rhythm, 1 (3%) had sinus bradycardia, and 2 (5%) had tachyarrhytmias with a moderate reaction to pharmacotherapy.

#### Pneumonia/sepsis

Pneumonia/sepsis developed in 9 of the 60 children (15.0%), 5 of 23 (22%) in Group HF and 4 of 37 (11%) in Group BDG, a statistically insignificant difference. The children who developed postoperative pneumonia/sepsis were intubated significantly longer than those who did not develop postoperative pneumonia/sepsis, both in Group HF (*p* = 0.025) and in Group BDG (*p* = 0.009).

#### Pre-and postoperative A-V valve regurgitation and single-ventricle heart function

Significant preoperative AVVR (Grade 2 or 3) was present in 12 (20%) of the 60 investigated patients, and postoperative AVVR was present in 7 (12%).

In Group HF, significant preoperative AVVR was present in 5 of 23 (22%) patients, of whom 4 (80%) had significant single-ventricle dysfunction. AVVR developed in 3 (13%) children with HLHS and 2 (8%) patients with unbalanced atrioventricular septal defect (UAVSD). Two (9%) children with HLHS had postoperative improvement of atrioventricular valve function, whereas 3 (13%) with this defect had no improvement (and died) - 2 (9%) of them developed arrhythmias, and 1 (4%) had fatal pneumonia/sepsis.

In Group BDG, 7 of 37 (19%) children had significant AVVR, shown in preoperative echocardiography; 5 (14%) had HLHS and 2 (5%) had UAVSD. Six (86%) patients with significant AVVR also had significant single-ventricle dysfunction. After bidirectional Glenn, echocardiography in 3 (8%) patients with HLHS revealed improvement in tricuspid valve competence, whereas no improvement was seen in the other 4 (11%); 2 (5%) of these patients developed arrhythmias, and 2 (5%) developed pneumonia/sepsis; these four children died.

The rates of postoperative single-ventricle heart function of grades 1–2 and 3–4 were similar in the two groups.

#### Mortality

Four of 23 (17.4%) children in Group HF died. Three (75%) had significant atrioventricular valve regurgitation before and after operation. Postoperatively, 2 of the patients (50%) developed arrhythmias – sinus bradycardia in 1 (25%) and slow sinus heart rhythm in the other (25%). None of the deceased children presented with tachyarrhythmia. One (25%) deceased child, with significant atrioventricular valve regurgitation, developed sepsis. In 1 (25%) child, sinus bradycardia occurred, and the patient died due to low cardiac output despite effective atrial pacing. The deceased patients in Group HF had significantly more frequent arrhythmias (75% vs. 11%; *p* = 0.02) and AVVR (75% vs. 11%; p = 0.02) than did the survivors.

Four for 37 (10.8%) children from Group BDG died. All had significant atrioventricular valve regurgitation. Two (50%) of the children also developed supraventricular tachyarrhythmia, and 2 (50%) had pneumonia/sepsis. The deceased patients In Group BDG had significantly more frequent arrhythmias (50% vs. 6%; *p* = 0.05), more frequent significant AVVR (100% vs. 9%; *p* < 0.001) and more frequent pneumonia/sepsis (50% vs. 6%; p = 0.05) than did the survivors.

#### Univariate and multivariate analysis of risk factors and death

**Fig. 2:**
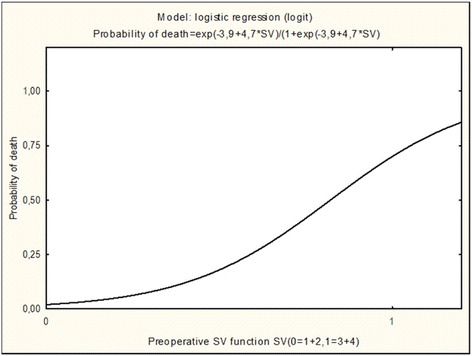
Following the stepwise elimination of the least significant factor in each step, SV (single ventricle) dysfunction was found to significantly affect mortality rates (*p* < 0.001)

Univariate analysis was performed to identify risk factors for death by patient characteristics and clinical and echocardiographic data (Table 3). Preoperative single-ventricle heart dysfunction (grade 3–4; significant impairment); preoperative clinically significant AVVR (grade 2–3; worse than mild); arrhythmias; and sepsis were associated with death. Multivariate analysis then was performed, with logistic regression in a model that took statistically significant preoperative variables from the univariate analysis. In the multivariate analysis, significant preoperative single-ventricle heart dysfunction was the only independent prognostic risk factor for death in second-stage palliation of single-ventricle heart anatomy (odds ratio 4.7; *p* < 0.001) (Fig. 2).

**Table 3:** Univariate analysis of risk factors for second-stage palliation of single-ventricle heart

Variables		Survived		Deaths		Total		*p* value
		n	%	n	%	n	%	
Preoperative SV function (0 = 1 + 2,1 = 3 + 4)	0	49	94.2	1	12.5	50	83.3	<0.001
	1	3	5.8	7	87.5	10	16.7	
Preoperative AVVR (0 = 0 + 1,1 = 2 + 3)	0	31	59.6	1	12.5	32	53.3	0.020
	1	21	40.0	7	87.5	28	46.7	
Arrhythmias	0	48	92.3	3	37.5	51	85.0	0.001
	1	4	7.7	5	62.5	9	15.0	
Postoperative sepsis	0	49	94.2	5	62.5	54	90.0	0.027
	1	3	5.8	3	37.5	6	10.0	
Total		52	100	8	100	60	100	–

## Discussion

Our study showed that clinically significant preoperative AVVR, preoperative single-ventricle dysfunction, pneumonia/sepsis, and arrhythmias were associated with increased mortality in patients with single-ventricle heart disease who underwent second-stage palliation. Only significant preoperative single-ventricle dysfunction was an independent prognostic risk factor. We did not find significant differences in clinical data, echocardiographic findings, or outcomes of patients treated with the hemi-Fontan compared with the bidirectional Glenn technique.

The mortality rate in our patients was higher than rates reported by centers treating larger numbers of patients [10]. This difference might be due to greater patient complexity in our population, as we aggressively accept high-risk patients. In recent years, the mortality rate in our patients from Group BDG has decreased compared with the rate in Group HF, even though Group BDG included older children with more complex defects than did Group I. A high percentage of our patients (20%) had significant AVVR, which has been reported to have a high perioperative risk [10, 12]. We emphasize that, except for one patient with complex heterotaxy syndrome, all children in our study who died had significant AVVR preoperatively; the significant AVVR persisted after operation, and the children also developed arrhythmias and/or pneumonia/sepsis.

Significant AVVR in our patients was due to dysfunction of the very valve and/or dysfunction of the single-ventricle. Disturbances of the structure/competence of the valve are most commonly associated with the anatomical background of the single ventricle heart and usually appear as unbalanced forms of common atrioventricular canal, as well as in heterotaxy syndrome. A study [10] has shown that even in the absence of associated defects and physiologic derangements, UAVSD confers risk on patients with single-ventricle heart. Mortality rates in these patients are high, regardless of whether valvuloplasty is performed in parallel with the second-stage palliation of single-ventricle heart [13]. In view of problems inherent in A-V valvuloplasty and the unimpressive results, we have not attempted this procedure. In cases of AVVR combined with ventricular dysfunction, we tried to perform stage II palliation at an earlier age (about 4 months of life), so the volume load of the single-ventricle could normalize earlier [1, 3]; with earlier operation, AVV competence was improved in children with HLHS but not in those with UAVSD, a result that is concordant with those of others [10].

In this study, significant single-ventricle dysfunction was an independent prognostic risk factor in patients with single-ventricle heart after second-stage palliation. Dysfunction of the single ventricle determines the occurrence of other complications that increase the risk of death in the early postoperative period. Bharucha et al. [14] demonstrated that right ventricular mechanical dyssynchrony and inhomogeneous contraction were worse in patients with clinically important tricuspid regurgitation and HLHS. Ventricular dysfunction leads to atrioventricular regurgitation, which in time results in progressive circulatory insufficiency. Patients with this insufficiency are susceptible to infections and often are operated on after numerous infectious episodes, when they are colonized by pathological bacterial and fungal flora.

Changing the surgical technique from the hemi-Fontan to bidirectional Glenn procedure did not significantly affect the prevalence of postoperative arrhythmias in our cohort. Similarly, as in other reports [15, 16], sinus bradycardia and slow sinus rhythm were the predominant postoperative cardiac rhythm abnormalities after second-stage palliation of single-ventricle heart. In 2 patients, supraventricular tachyarrhythmia developed after the bidirectional Glenn procedure, a complication that we believe has not been described. In each case of second-stage single-ventricle palliation, interatrial communication was enlarged surgically. On the one hand, such management allows for achieving wide interatrial communication, whereas on the other, it poses a threat of damaging the impulse conduction pathways between the sinus node and the sinoatrial node. Love et al. [17] showed that the atriotomy, right atrial free-wall scars, and atrial septal scars were predictors of tachyarrhythmias in patients after congenital heart diseases operations. Our patients with tachyarrhythmias had both atriotomy and atrioseptostomy, which we feel may have triggered arrhythmias.

For reconstruction of the pulmonary arteries and widening the SVC-pulmonary arteries anastomoses we have used an ECM (CorMatrix®) patch. This material has proven safe in reconstruction of low-pressure vessels. However, Hibino et al. [18] observed that at a mean follow-up of 9.7 months, 8 of 10 patients who underwent central pulmonary artery reconstruction with CorMatrix® tube had progressive significant stenosis. To avoid this complication, we used oversized anastomoses between the pulmonary arteries and the SVC.

There are limitations of this study. First, the number of the patients was small, although the study does have the advantage of uniform management in a single center. Second, the retrospective nature of the study could have introduced bias that affected comparisons between treatment groups. A prospective, randomized study of second-stage palliation (hemi-Fontan vs bidirectional Glenn procedure) of single-ventricle heart disease is needed. Third, in all patients, the ventricular function was evaluated semi-quantitatively. All patients were qualified to stage II on the base of echocardiographic examination; in some examinations it was impossible to visualize the size of all pulmonary arteries; thus, unfortunately, we could not include this important measurement in our analysis.

## Conclusions

Significant preoperative atrioventricular valve regurgitation, arrhythmias and sepsis are closely correlated with mortality in patients with single-ventricle heart disease after second-stage surgical palliation. Preoperative significant single-ventricle dysfunction is an important problem that has not been overcome by staged repair and has the highest impact on the mortality rate after second-stage surgical palliation. The bidirectional Glenn technique in surgical treatment of single-ventricle heart does not have a lower the incidence of early complications than does the hemi-Fontan operation.

## Abbreviations

AVVR: Atrioventricular valve regurgitation
DILV: Double inlet left ventricle
ECM: Extracellular matrix
HLHS: Hypoplastic left heart syndrome
SatO2: Oxygen arterial blood saturation
SVC: Superior vena cava
TA: Tricuspid atresia
UAVSD: Unbalanced atrioventricular septal defect
